# Supplementary material for: Sorting at embryonic boundaries requires high heterotypic interfacial tension
Source: Nat Commun. 2017 Jul 31;8:157. doi: 10.1038/s41467-017-00146-x (PMC5537356; doi:10.1038/s41467-017-00146-x)
Supplement: Supplementary file 2 — Supplementary Software 1 [file 41467_2017_146_MOESM2_ESM.zip › PottsModel/SrcPottsModel/doc/model/package-summary.html]

model


JavaScript is disabled on your browser.


Skip navigation links


- Overview
- Package
- Class
- Use
- Tree
- Deprecated
- Index
- Help

- Prev Package
- Next Package

- Frames
- No Frames

- All Classes

# Package model

- Interface Summary

  | Interface | Description |
  | --- | --- |
  |  |  |
  | --- | --- |
  | Coordinates |  |
  | EnergyFunction |  |
  | IEnergyFunction |  |
  | IPositionManager |  |
  | KnowsConstants |  |
  | Vector |  |
- Class Summary

  | Class | Description |
  | --- | --- |
  |  |  |
  | --- | --- |
  | AreaEnergyFunction |  |
  | CartesianCoordinates |  |
  | Cell |  |
  | CellSet |  |
  | Constants | Reads Constants from config file |
  | EnergyManager | Manages energy functions. |
  | FiniteVector |  |
  | HalfLine |  |
  | HexagonPositionManager |  |
  | InterfacialTensionEnergyFunction |  |
  | Lattice |  |
  | LatticeUtilities |  |
  | Pair<A,B> |  |
  | PerimeterEnergyFunction |  |
  | Pixel |  |
  | PixelInteractionEnergyFunction |  |
  | SquareCell |  |
  | SquarePositionManager |  |
  | VectorTests |  |
  | VectorUtils |  |
- Enum Summary

  | Enum | Description |
  | --- | --- |
  |  |  |
  | --- | --- |
  | Cell.CellType |  |
  | Cell.Energy |  |
  | Cell.State | Enum class representing the different states of the cell that can be passed as a notification message to the different class observers. |
  | Constants.Model |  |
  | EnergyFunction.Energy |  |
  | IPositionManager.Position |  |
  | Lattice.State |  |
  | Lattice.Type |  |
  | Pixel.PixelType |  |

Skip navigation links


- Overview
- Package
- Class
- Use
- Tree
- Deprecated
- Index
- Help

- Prev Package
- Next Package

- Frames
- No Frames

- All Classes
